# A reappraisal of the mucoactive activity and clinical efficacy of bromhexine

**DOI:** 10.1186/s40248-017-0088-1

**Published:** 2017-03-20

**Authors:** Alessandro Zanasi, Massimiliano Mazzolini, Ahmad Kantar

**Affiliations:** 1 Italian Association for Cough Study (AIST), Via Mazzini 12, 40138 Bologna, Italy; 2 Department of Specialistic-Diagnostic and Experimental Medicine (DIMES), Respiratory and Critical Care Unit, S.Orsola-Malpighi Hospital, University of Bologna, Via Massarenti 9, 40138 Bologna, Italy; 3 Pediatric Asthma and Cough Centre, Istituti Ospedalieri Bergamaschi, University and Research Hospitals, Via Forlanini, 15, 24036 Ponte San Pietro, Italy

**Keywords:** Bromhexine, Cough, Expectorants [Pharmacological Action], Bronchitis, Chronic, Pediatrics

## Abstract

Since its introduction to the market in 1963, bromhexine, an over-the-counter drug, has been investigated for its activity in animal models and in humans with diverse respiratory conditions. Bromhexine is a derivate of the *Adhatoda vasica* plant used in some countries for the treatment of various respiratory diseases. Bromhexine has been found to enhance the secretion of various mucus components by modifying the physicochemical characteristics of mucus. These changes, in turn, increase mucociliary clearance and reduce cough. Principal clinical research studies were primarily developed in an era when stringent methodological approaches and good clinical practices were not developed yet. Clinical studies were conducted mainly in patients with chronic bronchitis and in patients with various respiratory diseases, and demonstrated the efficacy of bromhexine in improving respiratory symptoms. Furthermore, the co-administration of antibiotics with bromhexine amplified the actions of the antibiotic. Although the clinical evidence shows only modest but positive results, bromhexine is indicated for its mucoactive activity. Larger trials with adequate methodology are required to identify when treatment with bromhexine can improve clinical outcomes.

## Background

Airway inflammation, mucus hypersecretion and impaired mucociliary clearance are the major characteristics of a variety of pulmonary diseases. Effective mucus clearance is essential for lung health, and airway disease is a typical consequence of poor clearance. Physiologically, mucus is a gel with low viscosity and elasticity that is easily transported by ciliary action, whereas pathologic mucus has higher viscosity and elasticity and is less easily cleared. Furthermore, beating cilia are in contact with the mucus layer and the contact is dependent, in part, on the rheology of the mucus. The transformation from healthy to pathologic mucus occurs by multiple mechanisms [1]. These mechanisms include modifications in the quality, quantity and physiochemical features of mucus.

Tracheobronchial secretions are principally watery (approximately 96% water) with approximately 4% mucus. The secretions form a layer over the ciliary epithelium, which consists of a fluid periciliary solution that enables the cilia to beat freely and propel the uppermost gel layer up to the airways [2].

Airway mucus is a heterogeneous mixture of secreted polypeptides, cells and cellular debris that is present in the fluid lining the airway surface subphase or is tethered together at the fluid surface by oligomeric mucin complexes [3]. Other locally synthesized macromolecules, including lysozyme, lactoferrin and immunoglobulins, are present in mucus. The total volume of secretions cleared per day in humans is not known, but it is estimated to be 10–50 ml [4]. This amount can increase up to 200 ml in individuals with chronic bronchitis during an exacerbation.

Elevated mucin production increases the number of intracellular mucin stores contained within airway secretory cells; in addition, increased mucin exocytosis increases the thickness and viscosity of the extracellular mucus gel positioned above the surface epithelium [3]. Numerous mucoactive agents with a variety of actions on the airways or secretions have been used. Patients who are most likely to benefit from mucoactive therapy usually have a history of increased sputum expectoration and a preserved airflow. However, the effectiveness of therapy in an individual patient can be difficult to assess [5].

*Adhatoda vasica* nees (Acanthaceae), commonly known as vasaka, is distributed throughout India up to an altitude of 1300 m. *Adhatoda vasica* is a medicinal plant native to Asia, widely used in Siddha, Ayurvedic and Unani systems of medicine [6]. *Adhatoda vasica i*s well known for its use in respiratory diseases. Both pure vasicine and its derivatives have been studied for their bronchodilatory and antitussive effects. Amin and Mehta were the first to isolate vasicinone, a component with bronchodilatory activity, in crystalline form from the leaves of *Adhatoda vasica* [7]. One of the derivatives of vasicinone is bromhexine (BHC) or bromhexine hydrochloride (N-cyclohexyl-N-methyl-(2-amino-3,5-dibromo-benzyl)amine hydrochloride). Since its introduction to the market in 1963, the drug has been investigated for its effects in animal models and in human with diverse respiratory diseases. In addition to clinical aspects, these studies have focused on the effects of the drug on mucus, sputum and mucociliary activity. In the present overview, we will highlight the principal basic findings about the mechanisms of action of BHC. In addition, we report a series of clinical findings in adults and children.

## Mechanisms of action

The mechanisms of action of BHC are complex and remain incompletely explored. To date, few has been reported regarding the cellular effects of the drug on the respiratory tract. Studies in animals and humans have reported actions that influence the production of mucosubstances, sputum quality and quantity, ciliary activity, antibiotic penetration and cough severity and frequency. These actions characterize the basic mucoactive activity of BHC and differentiate it from other drugs. Preclinical studies are summarized in Table 1.

**Table 1:** Summary of preclinical studies on bromhexine

Author	Cell type	BHC dosage	Outcomes
Merker 1966 [8]	Rats, bronchial secretory cells	4-20 mg/kg/24 h parenteral	Quantitative and qualitative effect on mucus production
Janatuinen 1969 [10]	Guinea pigs, bronchial goblet cells	5-10 mg/kg/24 h parenteral	Reduction in goblet cells, increased secretory material in airways
Gil 1971 [11]	Rats, type II alveolar cells	200 mg/kg/24 h enteral	Increased secretion of phospholipids in alveolar space
Harada 1977 [12]	Dogs, tracheal mucosa	4 mg intravenous	Increase in the output of secretion; goblet cells and submucosal mucus glands emptying
Von Wichert 1977 [14]	Rabbits, lung and liver tissues	10 mg/kg intravenous	Rapid stimulation of phospholipid synthesis
Martin 1993 [27]	Pigs, rheological characteristics of tracheal mucus	0,5 mg/kg	Increased concentration of oxytetracycline within the secreted mucus and reversed mucospastic activity of oxytetracycline in vivo

*BHC* bromhexine hydrochloride

### Effects of BHC on the production of mucosubstances

In 1966, Merker reported that the administration of BHC is followed by a quantitative and qualitative effect on secretory epithelial cells in the medium and small bronchi of the rat using electron microscopy [8]. In this pioneer study, BHC was injected at a dose of 4–20 mg/kg/24 hrs over a period of 2, 4 or 6 days. These observations were later confirmed in human tissue by Gieseking and Baldamus [9]. This later study was conducted on 9 patients with tuberculosis with cavitation or carcinoma before and after partial lung resection. Biopsy samples were taken before resection and BHC was given at a dose of 4 mg three times daily. BHC considerably increased secretion activity in the seromucous glands of the bronchial mucosa. The secretion product of the glandular serous epithelial cells consisted of large homogeneous globular granules. These granules exhibited ultrastructural characteristics that were strikingly similar to lysosomes. No quantitative changes were observed in ciliated or goblet cells.

Janatuinen and Korhonen evaluated the effect of BHC on mucosubstance production in 15 male guinea pigs [10]. Animals were divided into 5 groups: control, treated with saline, treated with 10 mg/kg BHC intraperitoneally, treated with 10 mg/kg BHC subcutaneously for 3 day and treated with 5 mg/kg BHC subcutaneously for 7 days. Samples from the trachea, salivary glands and duodena were examined to determine the number of goblet cells and for histochemical staining for mucosubstance investigations. The secretory material in tracheobronchial goblet cells consisted of acid mucosubstances containing both sulphate and carboxyl groups in addition to periodate reactive material. Drug treatment induced changes in the synthesis of acid mucosubstances, resulting in secretory material in the tracheal mucosa that consisted largely of neutral periodate reactive substances. Higher doses of BHC caused a significant decrease in the total number of goblet cells with secretory material, especially the cells containing sulphated mucosubstances. Authors attributed these findings to the increased mobilization of the secretory products from the cells. The drug did not induce changes in the salivary glands or in the duodenal goblet cells.

Gil and Thurnheer evaluated the effect of BHC on the ultrastructure of type II alveolar cells obtained from white rat lungs [11]. In this study, the drug was administered through a gastric tube (200 mg/kg/day) for 3 days. The results showed that type II alveolar epithelial cells presented with lamellated bodies of unusual size, and many cells were “stuffed” with lamellated bodies in the BHC group. The morphometric evaluation indicated a volumetric density of lamellated bodies within type II alveolar cells of 20 ± 0.6% in the treated group with BHC and 18 ± 0.8% in the control group. The volumetric density of alveolar type II cells within the lung was 11.3 ± 1.5% in the treated group and 6.8 ± 1.8% in the control group. These data indicate a considerable increase in the number of lamellated bodies in lung parenchyma from 1.22% in controls to 2.26% in the BHC group. Alveolar type II cells are considered to be major sources of alveolar surfactant. This study indicates that BHC influenced the metabolism of alveolar type II cells, which might lead to an increase in the secretion of phospholipids into the alveolar space.

Harada et al. evaluated the effect of an intravenous injection of 4 mg of BHC on tracheal mucosa secretory activity in adult dogs [12]. The secretory activity was evaluated by observing ultrastructural mucosal changes after 15, 30 and 60 min. using electron-microscopy scanning. Drug administration induced a rapid increase in the output of secretion from normal tracheal mucosa. Both goblet cells of the epithelium and the sero-mucinous glands in the tunica propria participated in the production of the tracheal discharge. Two types of secretions were observed: spherical mucus granules derived from goblet cells and submucosal mucus glands; and a fine floccular substance that stuck the tips of cilia together and appeared to be serous discharge or transudate. Sixty minutes after drug injection, non-granules were observed on the epithelial surface and the submucosal glands were shrunken and empty. These data demonstrate an active action of BHC on tracheal cells and consequently on mucociliary clearance.

Lorenz et al. demonstrated that the administration of the BHC metabolite VIII to 36 pregnant women with a gestational age of 29–32 weeks at a dose of 1 mg/kg/day for 5 days induced fetal surfactant production [13]. Compared to controls, an increase of more than 50% in total phospholipid concentration, lecithin concentration and lecithin/sphingomyelin ratio was observed. The administration of BHC at doses of 300–600 mg orally daily or 100 mg intravenously for five days did not induce the previously mentioned changes. The authors attributed this finding to the low dose of BHC. The incorporation of lauric acid, palmitic acid and oleic acid into phospholipids of lung and liver tissues of control rabbits and rabbits treated intravenously with BHC or ambroxol at 10 mg/kg was investigated by Von Wichert et al. using radioactively labelled precursors [14]. A marked increase (up to 200%) in palmitic acid incorporation into phosphatidylcholine (lecithin) and phosphatidyl-ethanolamine in the lung was observed but was not present in the liver. The observed effects were more marked in shorter experiments (analysis 2 hrs after drug injection) than after treatments for 7 days. The incorporation of lauric acid and oleic acid into lung phospholipids was not influenced by treatment. These data demonstrate that BHC stimulates phospholipid synthesis.

Crimi et al. investigated the effect of 15 days of treatment with BHC 48 mg/day on phospholipids in bronchoalveolar fluid in 13 patients suffering from chronic bronchitis [15]. After treatment, the concentration of total phospholipids significantly increased (*p* < 0.05). This increase was not associated with variations in the phospholipid fractions. The authors hypothesized that this increase may be attributed to an increase in the phospholipid content of type II cells or Clara’s cells or a decrease in the degradation of phospholipids. These changes may enhance mucociliary clearance in the lung.

Membrane phospholipids constitute a permeability barrier, modulate the functional properties of membrane-associated activities, provide a matrix for the assembly and function of a wide variety of catalytic processes, and act as donors during the synthesis of macromolecules [16]. Furthermore, peroxidation of membrane phospholipids acyl chains produces agents that play a causal role in various pathological process [16, 17].

### Effect of BHC on sputum

Flavell Matts et al. investigated the effect of BHC on sputum fiber systems during exacerbations of pulmonary disease in 53 patients between 15 and 86 years old with asthmatic bronchitis and acute or chronic bronchitis [18]. Morning sputum was collected three times weekly and fixed smears were stained and observed under fluorescent and polarized light to examine DNA or mucopolysaccharide (MPS) fiber systems, respectively. Thirty-one patients were treated with antibiotics and BHC, 14 patients were treated with BHC, only and 8 patients with antibiotics only. BHC was administered at a dose of 8–32 mg 3 times daily. The results demonstrated that when patients responded to antibiotic treatment, DNA fibers disappeared within a week. However, in some patients, the amount of sputum was not reduced after antibiotic therapy even after DNA fibers disappeared; this was due to the presence of MPS fibers. In patients treated with BHC, the MPS fibers usually disappeared within 7–12 days (average 10 days). Moreover, patients in the BHC group demonstrated clinical progress in that they were more able to expectorate and sputum production markedly decreased. The authors concluded that antibiotics did not affect the MPS fibers, but BHC therapy could reduce or entirely remove these fibers from the sputum.

In a single-blind study, Bürgi evaluated the effect of BHC and guaifenesin on the fiber system and the viscosity of sputum in 22 adult patients with chronic bronchitis [19]. Seventeen patients completed the cross-over study. For 2 weeks, the first group of 8 patients were allocated to receive 3x8 mg BHC syrup daily. Then, subjects received 3x100 mg of guaifenesin syrup for an additional 2 weeks. The remaining patients received treatments in the reverse order. Glycoprotein (GP) fibers in sputum and sputum viscosity were evaluated at weekly intervals in all patients. Investigations were carried out with a rotational Viscotester under thermostable conditions with a constant shear rate of 180 s^−1^. The results demonstrated that treatment with BHC progressively reduced the fiber content of sputum, which increased again after subsequent treatment with guaifenesin. Irrespective of the treatment order, BHC was clearly superior with regard to the effects on GP fibers (*p* < 0.01). Sputum viscosity decreased significantly in the BHC group (*p* < 0.01). It is believed that a reduction in sputum fiber content decreased viscosity.

The effects of various drugs, including BHC, on sputum viscoeleasticity in 40 adults with chronic pulmonary diseases (excluding asthma) were investigated by Shimura et al. [20]. Patients were allocated into 5 groups: a control group, a BHC group (24 mg/day), an ambroxol group (90 mg/day); an α-chymotrypsin group (100 cu.u/day administered total cavity) and a serratiopeptidase group (30 mg/day). The treatment lasted for one week. Viscoelastic measurements were obtained with a Raised Cosine Pulse (RCP) method using a coaxial cylinder rheometer. The RCP strain with a frequency of ωο 1.05 rad.sec ^−1^ was added by applying one cycle oscillation with an amplitude of 5° to the outer cylinder and the Fourier transformation of this stress was performed to obtain a complex shear modulus (G’: storage modulus and G”: loss modulus). The results demonstrated that absolute values of G’ and G” at a frequency of ω5x10^−1^rad.sec^−1^ before and after the treatment were significantly decreased (0.005 < *p* < 0.01) only in the BHC group. In the BHC group, the frequency dependence of G’ and G" remained unchanged. On the other hand, chymotrypsin and serratiopeptidase (proteolytic enzymes) markedly changed the frequency dependence of the sputum on G’ and G”. These data indicate that BHC does not influence the molecular structure of the mucus, whereas the aforementioned proteolytic enzymes break down the linkage between structural subunits of the sputum.

In contrast to these data, a study by Langlands [21] did not report any significant changes after BHC treatment. In this study, the effects of BHC were compared with those of placebo in a double blind clinical trial in patients with exacerbations of chronic bronchitis who also had mucoid sputum. Treatment with either BHC 8 mg three times a day or with identical placebo tablets was continued for 14 days. There was no significant effect on the characteristics of the sputum, the improvement in ventilatory capacity, or clinical characteristics in patients who had been treated with BHC. Details of the study are reported later. Similarly, a report with a higher dose of BHC was published later [22]. It stated that the effects of 48 mg of BHC daily for 2–3 weeks were indistinguishable from those of placebo tablets with respect to the stickiness of sputum, difficulty of expectoration, or time taken to clear the chest in the morning. Reasons behind these contrasting results are discussed later.

Sputum samples of 20 patients with allergic asthma and the sputum pool of 10 healthy men were analyzed before and after the administration of BHC [23]. BHC was administered at the following doses: 3x8 mg from the first to the 3^rd^ day, 2x8 mg on the 4^th^ and 5^th^ days and finally 4x4 mg from the 6^th^ day onward until the 10^th^ day. Sputum samples were characterized by electrophoresis using biochemical and immunological methods. After treatment all primary demonstrable protein components showed a marked increase, especially gamma globulins. This rise was interpreted as the result of increased secretion of already synthesized immunoglobulin molecules. Similar observations were reported by Kado, who confirmed increased concentrations of IgA and IgG in bronchial washings after BHC treatment in 13 patients with chronic bronchial infections [24].

### Effect on ciliary clearance

Thomson et al. investigated the effect of BHC on mucociliary removal rate in 9 adult subjects with chronic bronchitis (simple, mucopurulent and obstructive) [25]. Each patient was treated for 14 days with 16 mg of BHC three times daily. Mucociliary removal activity was evaluated using labelled aerosol (99mTc) after inhaling a dose of <50 μCi. Clearance of the radioactivity was faster in the patients than in the normal group. The amount cleared at 6 h increased by 6.8% after treatment with the drug (*p* < 0.05), which represents an increase of 14.5% over radioactivity cleared in the control run. Scanning of the lung after inhalation showed that tracer particles penetrated further and were deposited nearer to the periphery of the lung after BHC treatment than after control treatment and the differences were statistically significant (*p* < 0.01).

Pavia et al. evaluated the effect of BHC and other expectorant agents on tracheobronchial clearance in 43 patients with chronic bronchitis using a radioaerosol tracer technique [26]. Following the inhalation of the radioaerosol under strictly controlled conditions, its clearance from the lungs by means of the mucociliary system and productive cough were monitored for 6 h. Treatment with bromhexine, like other drugs (guaifenesin, 2-mercapto-ethane sulphonate and hypertonic saline 1.21 M), significantly enhanced lung clearance.

### Effect of BHC on antibiotic penetration

Martin et al. investigated the influence of the combined administration of BHC and oxytetracycline hydrochloride on the rheological characteristics of tracheal mucus in three adult mini-pigs [27]. A twice-daily dosage of oxytetracycline (40 mg kg − 1) and BHC (0.5 mg kg − 1) was employed. Mucus was collected daily from open-ended tracheal pouches established surgically in the mini-pigs. The viscoelastic properties of each mucus sample were determined using creep compliance analysis in a rheometer. The results showed that oxytetracycline increased the residual shear viscosity (*p* < 0.01) and the instantaneous compliance (*p* < 0.01). When BHC was co-administered with oxytetracycline, all of these changes were abolished. Data suggest that BHC increased the concentration of oxytetracycline within the secreted mucus and reversed the mucospastic activity of oxytetracycline in vivo.

Bergogne-Berezin et al. evaluated the influence of BHC on the penetration of erythromycin into bronchial secretions [28]. In a double blind placebo controlled study 22 patients who had undergone bronchoscopy received either erythromycin (day 0: 0 mg; day 1: 1000 mg; day 2: 1500 mg, day 3: 500 mg) and placebo or erythromycin (same as the control group) together with BHC (day 0: 8 mg; day 1: 12 mg; day 2: 12 mg; day 3: 4 mg). Eighteen patients completed the study. Erythromycin concentration was determined in serum and in bronchoalveolar fluid collected by bronchoscopy after treatment. In the BHC group, the antibiotic concentration was 0.61-3.2 μg/ml (mean 1.55 μg/ml), whereas in the placebo group the antibiotic concentration was 0.4-1.6 ug/ml (mean 1.05 μg/ml). The mean ratio of bronchial/serum level of erythromycin in the BHC group was 0.46 vs 0.25 μg/ml in the control group (*p* = 0.05). This study revealed a significant increase in the ratio between bronchial levels and serum concentrations of erythromycin when erythromycin was administered in combination with BHC. These data suggest that BHC enhances erythromycin concentration in bronchial fluid.

Later clinical studies verified that combination treatment with BHC and antibiotics was associated with favorable clinical responses. A multicenter double blind trial by Roa and Dantes reported a significant reduction in symptoms such as cough discomfort, cough frequency, ease of expectoration and sputum volume in the group treated with amoxicillin plus BHC with respect to the group treated with amoxicillin alone [29]. Details of these studies are reported below.

### Cough

Various clinical studies have demonstrated that BHC influences cough. Lal and Bhalla demonstrated that treatment with BHC leads to a reduction in expectoration and reduced the severity/frequency of coughing [30]. The alleviation of cough and the normalization of expectoration have been described in patients with chronic obstructive lung disease in a study by Iaia and Marenco [31]. Details of these and other observations are described in the subsequent clinical studies section.

## Efficacy and safety of bromhexine in adults

Studies in animal models have raised clinical interest in BHC. Numerous studies in adults with mucus hypersecretion have subsequently been conducted. Herein we analyze the fundamental studies present in the literature (Table 2). Most of these studies were performed in subjects with chronic obstructive bronchitis.

**Table 2:** Summary of clinical study on bromhexine

Author	Study design	Subjects	BHC dosage	Outcome	Adverse events (n)
Gieseking 1968 [9]	Observational	Biopsy from 9 adult patients with cavitated tubercolosis or carcinoma	4 mg 3 times/24 h	Increased secretion activity in the seromucus glands of the bronchial mucosa	-
Lorenz 1974 [13]	Observational	36 pregnant women	1 mg/kg/24 h	Increase > 50% in total phospholipid concentration in fetal surfactant	-
Crimi 1986 [15]	Observational	13 adult patients with chronic bronchitis	48 mg/24 h	Increase concentration of total phospholipids in bronchoalveolar fluid	-
Flavell Matts 1973 [18]	Observational	53 adult patients with asthmatic bronchitis and acute or chronic bronchitis	8-32 mg/3 times/24 h	Reduced mucopolysaccharide fiber systems in sputum	-
Bürgi 1974 [19]	Single-blind crossover	22 adult patients with chronic bronchitis	8 mg/3 times/24 h	Reduction in sputum glycoprotein fiber content and decreased viscosity	-
Shimura 1983 [20]	Observational	40 adults with chronic pulmonary diseases (excluding asthma)	24 mg/24 h	BHC does not influence the molecular structure of the mucus	-
Langlands 1970 [21]	Double blind randomized controlled trial	27 adult patients with exacerbations of chronic bronchitis	8 mg/3 times/24 h	No significant difference in respiratory function or mucus properties	Nausea (*n* = 1)
Stark 1973 [22]	Randomized controlled trial	42 adult patients with chronic bronchitis	48 mg/24 h	No significant difference in mucus characteristics	-
Götz 1970 [23]	Observational	20 adult patients with allergic asthma	16-24 mg/24 h	Increased gamma globulins in sputum	-
Kado 1976 [24]	Observational	13 adult patients with chronic bronchial infections	-	Increased concentrations of IgA and IgG in bronchial washings	-
Thomson 1974 [25]	Observational	9 adult subjects with chronic bronchitis	48 mg/24 h	Increased mucociliary clearence	-
Pavia 1979 [26]	Observational	43 adult patients with chronic bronchitis	-	Increased mucociliary clearence	-
Bergogne-Berezin 1979 [28]	Double blind placebo controlled stud	22 adult patients undergone bronchoscopy and received erythromycin	4-12 mg/24 h	Increased erythromycin concentration in bronchial fluid	-
Roa 1995 [29]	Double blind multicenter randomized controlled trial	392 adult patients hospitalized for uncomplicated bacterial lower respiratory tract infections receiving amoxicillin	32 mg/24 h	Better overall resolution of symptoms and cough; increased expectoration	Undefined (*n* = 6)
Lal 1975 [30]	Randomized crossover placebo-controlled trial	41 adult patients with stable chronic obstructive bronchitis receiving oxytetracycline	48 mg/24 h	Better subjective evaluation of sputum stickiness and physician assessment of outcome	Headache (*n* = 2) Stomacache (*n* = 2)
Nesswetha 1967 [32]	Double blind randomized controlled trial	242 adult patients with mixed respiratory conditions	15 mg/24 h	Reduced cough	No adverse events reported
Gent 1969 [33]	Cross sectional, double blind, placebo controlled study	48 adult patients with chronic bronchitis, asthma, emphysema or diffuse parenchymal lung disease	24 mg/24 h	Overall clinical and functional improvement	Diarrhea (*n* = 1) Headache (*n* = 1)
Hamilton 1970 [34]	Double blind randomized controlled trial	22 adult patients recovering from an exacerbation of chronic obstructive bronchitis	48 mg/24 h	increased sputum production and reduced sputum viscosity	No adverse events reported
Christensen 1970 [35]	Double blind randomized controlled trial	61 adult patients with chronic bronchitis	24 mg/24 h	Better overall clinical improvement and increased FEV1	-
Condie 1971 [36]	Single blind randomized controlled trial	31 adult patients with chronic bronchitis	24 mg/24 h	Reduced symptoms, increased sputum volume and PEFR	Nausea and abdominal distension (*n* = 3)
Matts 1974 [37]	Double blind randomized controlled trial	102 hospitalized adult patients with lower respiratory tract infections treated with oxytetracycline	32 mg/24 h	Higher rate of favorable response to treatment, faster recovery and shorter hospitalization stay	Nausea and anorexia (*n* = 22)
Armstrong 1975 [38]	Randomized crossover placebo-controlled trial	12 adult patients with chronic bronchitis	72 mg/24 h	Increased expectoration, improved auscultatory findings and PEFR	Headache and nausea (*n* = 1) Dizziness (*n* = 1)
Valenti 1989 [39]	Double blind multicenter randomized controlled trial	237 adult patients with chronic obstructive lung disease	60 mg/24 h	decrease in cough, dyspnea and sputum volume; easier expectoration; improved auscultatory findings and improved FEV1 and PEFR; higher rates of treatment efficacy	Vomiting and gastralgia (*n* = 1)
Bienvenido 1990 [40]	Randomized controlled trial	28 adult patients with acute bronchitis or exacerbation of chronic bronchitis receiving amoxycillin	24 mg/24 h	Reduced symptom severity and higher bacterial elimination	-
Olivieri 1991 [41]	Double blind multicenter randomized controlled trial	88 adult patients with exacerbation of bronchiectasis	90 mg/24 h	Improved cough, auscultatory findings, expectoration difficulty and FEV1	-
Aylward 1973 [42]	Double blind multicenter randomized controlled trial	38 adult patients with chronic obstructive bronchitis	48 mg/24 h	Reduced sputum viscosity and increased expectoration volume	Gastrointestinal (*n* = 1)
Barth 2015 [43]	Randomized double blind controlled parallel trial	177 adult patients with cough due to uncomplicated upper respiratory tract infections	24 mg/24 h	Better cough relief	Mixed (pruritus, diarrhea, abdominal pain, skin rash) (*n* = 6)
Tarantino 1988 [44]	Double blind randomized controlled trial	30 children with acute sinus inflammation receiving amoxycillin	48 mg/24 h	Reduced nasal secretions, improvement in rhinitis; less school days lost	No adverse events reported
Molina 1970 [45]	Observational	48 infants with pharyngo-bronchitis, bronchopneumonia, bronchopneumonia with tubercolosis and asthmatic bronchitis	4-24 mg/24 h	Better overall clinical improvement	-
Fernandes 1973 [46]	Observational	30 children with clinical symptom of mucus retention (asthma, common cold and bronchiolitis)	0.5 mg/kg	Overall clinical improvement in patients with asthma and common cold	No adverse events reported
Brezina 1973 [47]	Observational	45 children with bronchitis	-	Overall clinical improvement	No adverse events reported
Okamoto 1981 [48]	Observational	37 children with bronchitis, common cold, asthmatic bronchitis, asthma and bronchiectasis	0.4-0.6 mg/kg	Overall clinical improvement	No adverse events reported
Koga 1981 [49]	Observational	32 children with upper respiratory tract inflammation, acute bronchitis, bronchopneumonia and asthma	0.4 mg/kg/3 times/24 h	Improved expectoration	No adverse events reported
Camurri 1990 [50]	Open randomized comparative study	32 children hospitalized for acute bronchitis	24 mg/24 h	Improved expectoration and clinical outcom	No adverse events reported
Azzolini 1984 [51]	Open randomized comparative study	40 children with hypersecretory bronchopulmonary diseases (acute, asthmatic or recurrent bronchitis)	6-12 mg/24 h	Improved general clinical conditions, dyspnea and sputum viscosity	Nausea and regurgitation (*n* = 2) Diarrhea (*n* = 1)
Boner 1984 [52]	Observational	100 children with respiratory tract infections	0.6-0-8 mg/kg/24 h	Overall clinical improvement and better resolution in patients with acute episodes	Gastric intolerance (*n* = 3)

*IgA* immunoglobulin A, *IgG* immunoglobulin G, *FEV*_*1*_ forced expiratory flow 1st second, *PEFR* peak expiratory flow rate, *BHC* bromhexine hydrochloride, − = not available

### Randomized controlled trials

Nesswetha [32] enrolled 242 patients with mixed respiratory conditions (chronic and acute bronchitis, colds and upper respiratory tract infections) in three different double blind randomized controlled trials. Participants received BHC 5 mg three times daily (tid) or placebo for at least four days. Clinical endpoints (cough, sputum viscosity, disability) showed that frequent cough (every two to five minutes) was less prevalent in the active treatment arm (8.6%, *p* < 0.02) compared to the placebo group (15.2%). The drugs were well tolerated, without any significant adverse effects reported.

Gent et al. [33] described the effects of BHC on pulmonary function in a cross sectional, double blind, placebo controlled study that included 48 patients (19–64 years old) with chronic bronchitis (*n* = 23), asthma (*n* = 9), emphysema (*n* = 8) and diffuse parenchymal lung disease (*n* = 8), presenting with difficulty in the expectoration of sputum. Each condition was diagnosed on the basis of the current diagnostic criteria. The measured outcomes were functional residual capacity (FRC), forced expiratory volume in one second (FEV_1_), total-body plethysmography, and clinical improvement. Outcomes were measured before the start of the first treatment, at the transition of the treatment arm and at the end of the observation period. After the randomization and the first week without treatment, patients received BHC tablets 8 mg three times a day (tid) or placebo for one week. In the third week of the study, subjects received the other treatment. The group treated with BHC had a significantly higher number of patients showing clinical and functional improvement. FRC and FEV_1_ measures were not significantly different between the two groups. The adverse effects of BHC included diarrhea (*n* = 1) and headache (*n* = 1).

Langlands [21] tested the effectiveness of BHC in patients admitted to the hospital for the exacerbation of chronic bronchitis. Twenty-seven patients were randomized to receive bromhexine 8 mg tid or placebo for 2 weeks. The measured outcomes included respiratory function (FEV_1_; forced vital capacity, FVC; peak expiratory flow rate, PEFR). Sputum viscosity and sputum volume were also compared. The authors did not find any statistically significant difference in respiratory function or mucus properties. One patient treated with BHC complained of nausea.

In 1970 Hamilton and colleagues [34] explored the rheological mucus and pulmonary functional effects of BHC in patients recovering from an exacerbation of chronic obstructive bronchitis. Twenty-two patients (age 53.2 ± 8.9 years) were randomized in a double blind manner to receive placebo (*n* = 10) or BHC (*n* = 12) 16 mg thrice daily for 11 days after an exacerbation of chronic bronchitis. Outcomes were measured at the beginning of the study, daily during the trial and four days after the end of the treatment. Functional outcomes included FEV_1_ and FVC, measured with a forced expiratory spirogram. Mucus viscosity and daily sputum volume were measured to assess rheological effects of BHC. The same physician performed the clinical assessment at the beginning and at the end of the study. The results showed a statistically significant increase in sputum production (*p* < 0.01) and a significant reduction (*p* < 0.01) of sputum viscosity in patients receiving BHC. Neither spirometry values nor clinical improvement were statistically significant between the groups. BHC was well tolerated, and did not exhibit significant hemato-chemical alterations.

In the same year, Christensen et al. [35] performed another randomized controlled trial in patients with chronic bronchitis (61 patients, aged 55.4 years). Sixty-one patients with a diagnosis of chronic obstructive bronchitis were divided into three groups based on disease severity and received BHC 24 mg daily or placebo for six months. Mucolytic activity was evaluated by assessing the variation of functional (FEV_1_; VC, vital capacity; PEFR) and clinical (days of illness, antibiotic consumption and subjective assessment) outcomes during the study period. The FEV_1_ was significantly different (*p* = 0.01) in patients with moderate disease. Clinical endpoints indicated that a greater proportion of patients felt better and rates of illness were lower among those who took BHC. Safety data were not reported.

The single blind study by Condie [36], performed in a mining area during the winter period, included 31 patients with chronic bronchitis who received BHC 8 mg three times daily or placebo for six weeks. The effects of treatment were evaluated on both clinical (symptoms, sputum characteristics) and functional parameters (PEFR). The results showed a subjective reduction of symptoms and an increased sputum volume in individuals who had taken BHC; PEFR was significantly increased after six weeks of treatment (*p* < 0.05). Three patients complained of gastrointestinal side effects (nausea and abdominal distension).

Matts conducted a double blind randomized trial on 102 hospitalized patients (mean age: 52 years) with lower respiratory tract infections (chronic bronchitis exacerbation, pneumonia, acute bronchitis) [37]. Both groups were treated with oxytetracycline 250 mg 4 times daily and one arm also received BHC 8 mg every 6 h for 10 days. Endpoints included the overall response to treatment as assessed by clinical and radiological improvements (rated as good, moderate or failed) and length of stay in the hospital. Sixty-seven percent of patients who had received BHC exhibited a favorable response to treatment, compared to 51% of patients who had received antibiotics only (p > 0.05). In addition, patients treated with BHC exhibited a faster recovery and a significantly shorter hospitalization stay (9.4 vs 11.2 days, *p* < 0.001). Twenty-two patients in the BHC group and 21 patients in the oxytetracycline alone group reported side effects that were primarily associated with the gastrointestinal system (nausea and anorexia).

Lal and Bhalla [30] studied the effect of BHC 16 mg 3 times daily in patients with stable chronic obstructive bronchitis in a randomized crossover placebo-controlled trial. Out of the 41 patients enrolled (mean age: 55.9 years) only 36 completed the 8 week study. Outcomes were clinical (subjective symptoms, physician evaluation) and functional (FEV_1_; PEFR; VC). All patients received oxytetracycline 500 mg twice a day during the study. The patients who received BHC exhibited better outcomes in terms of the subjective evaluation of sputum stickiness (*p* < 0.05) and the physician assessment of outcome (*p* < 0.05). No significant differences were detected in pulmonary function tests. Two patients complained of headache and two patients reported experiencing stomach pain during treatment with BHC.

In 1975, Armstrong conducted another randomized crossover placebo-controlled trial of BHC 24 mg tid for 4 weeks in 12 patients with chronic bronchitis [38]. Only ten subjects completed the 10 week trial. The effects of BHC were evaluated with functional measures (FEV_1_, FVC and PEFR) and clinical outcomes (sputum characteristics, auscultatory findings, dyspnea, cough, subjective ease to expectoration). The results demonstrated that patients who were treated with BHC exhibited increased expectoration (*p* < 0.05) associated with improved auscultatory findings (*p* < 0.004). PEFR was significantly improved (*p* < 0.02) in patients receiving BHC. Two patients reported adverse effects (1 patient reported headache and nausea, 1 patient reported dizziness).

An Italian multicenter randomized controlled trial by Valente [39] and colleagues evaluated the efficacy of BHC in patients with chronic obstructive lung disease. Two hundred and thirty-seven outpatients were selected from 7 Italian clinical centers and were randomized in double blind manner to receive either placebo or BHC 30 mg twice a day for 14 days. The authors evaluated sputum volume and quality, symptoms (expectoration, cough, dyspnea, and auscultatory thoracic symptoms), FEV_1_, PEFR and residual volume (RV) as clinical and functional outcomes. Patients treated with BHC showed a statistically significant decrease in cough and sputum volume, easier expectoration, improved auscultatory findings and decreased dyspnea (*p* < 0.01) compared to patients who had received a placebo. FEV_1_ and PEFR were significantly improved in patients treated with BHC in comparison to patients who received a placebo (*p* < 0.01). The results of the clinical assessment demonstrated higher rates of treatment efficacy in the treatment arm (*p* < 0.01). The treatment was well tolerated with only one case of withdrawal due to vomiting and gastralgia. No hemato-chemical alterations were observed.

Bienvenido D. Alora [40] evaluated the effects of dual therapy with BHC and amoxicillin in patients with acute bronchitis or an exacerbation of chronic bronchitis. Twenty-eight patients (14 with acute bronchitis and 14 presenting with an exacerbation of chronic bronchitis) were enrolled in the study; patients were randomized to receive amoxicillin alone (*n* = 13) or amoxicillin plus BHC (*n* = 15). Endpoints included a reduction in the nominal scale of symptoms and sputum volume. Patients who had received BHC demonstrated a reduction in symptom severity (*p* < 0.001) and bacterial elimination was higher than in subjects treated with amoxicillin alone.

In 1991, Olivieri and colleagues [41] studied the effects of BHC in patients with radiological evidence of bronchiectasis and a productive cough during an exacerbation. The study was a double blind, randomized, multicenter study including 88 patients who received BHC 30 mg three times a day or placebo three times a day for 15 days. A ceftazidine 1 g intramuscular injection was administered daily for the first week. The outcomes included the clinical evaluation of cough, auscultatory findings and expectoration difficulty using an arbitrary four-point scale and FEV_1_ measurements. Both clinical and functional outcomes were improved in patients who had been treated with BHC.

More recently, Roa and Dantes [29] published a double blind multicenter randomized controlled trial on patients admitted to the hospital for uncomplicated bacterial lower respiratory tract infections (LRTIs). Subjects with susceptible bacteria according to sputum culture were randomized to receive amoxicillin (250 mg 4 times/day, *n* = 200, mean age of 32 years old) or amoxicillin plus BHC (250 mg + 8 mg 4 times/day, *n* = 192, mean age of 32 years old) for 5 to 7 days. The endpoints consisted of the clinical resolution of the episode of LRTI; subjective dyspnea, cough and expectoration measured with a visual analog scale (VAS); and the eradication of the bacterial infection. Patients treated with a combination of amoxicillin and bromhexine exhibited a better overall resolution of cough (*p* < 0.001) and increased expectoration in the first day of treatment (*p* < 0.001) and a greater decrease of symptoms in the first days of treatment.

### Comparative studies

Aylward [42] explored the expectorant effects of s-carboxymethylcysteine and BHC in patients with chronic obstructive bronchitis in a double blind comparison study. Thirty outpatients (age 56.2 years) with a recent exacerbation were randomized to receive BHC 16 mg or s-carboxymethylcysteine 750 mg thrice a day for ten days. Both groups reported a reduced viscosity of sputum associated with an increased expectoration volume. No significant differences were detected in pulmonary function tests (FEV_1_, FVC, and PEFR). Two patients (One in each group) reported that they had experienced gastrointestinal adverse effects.

A recent randomized double blind parallel trial [43] compared BHC to a phytomedicine syrup (KJ) and placebo. One hundred and seventy-seven patients, 18 to 65 years old, with cough due to uncomplicated upper respiratory tract infections randomly received BHC (24 mg/30 ml/day, *n* = 57), KJ syrup (30 ml/day, *n* = 66) or placebo (*n* = 54) over a 5 day period. The primary outcome was the reduction of cough frequency from baseline assessed with a nominal 9 grade score. Both BHC and KJ syrup were more effective than placebo for cough relief. BHC showed a slightly longer latency of effect. Six patients in the BHC group reported minor adverse events, which the authors did not treat.

## Efficacy and tolerability of BHC in children

A substantial number of studies have investigated the efficacy and tolerability of BHC in children. Most of the studies were observational and empiric. Furthermore, they were conducted when the drug was introduced to the market. Almost all of these studies were based on clinical evaluations and lacked objective methods for the investigation of drug efficacy. Different formulations of the drug were employed. No double blind studies were conducted with the drug alone and sample sizes in these studies were small. Patients mainly presented with upper respiratory tract infections and studies were conducted with BHC alone or in association with antibiotics (see Table 2).

### Randomized controlled studies

A unique study conducted by Tarantino et al. investigated the advantages of treatment with BHC (16 mg 3 times/day for 8 days) in 30 children (age range 3.5-12 years) with acute sinus inflammation in a placebo-controlled randomized study [44]. Amoxicillin was administered orally to both groups of patients (50 mg/kg/day) three times a day for the entire study period. Therapeutic efficacy was evaluated by monitoring signs and symptoms of acute sinus inflammation (temperature, pain, rhinitis, nasal secretions and hyperemia) and by X-rays of the paranasal sinuses. Diagnostic X-ray images were used as inclusion criteria and were controlled 30 days later. An arbitrary scale from 0 to 4 (0 = absent, 1 = slight, 2 = moderate, 3 = intense, 4 = very intense) was used to evaluate pain, nasal secretions and rhinitis. Evaluations were performed on days 2, 4, 6 and 8 of treatment. The results demonstrated a significant reduction of nasal secretions (BHC group 0.06 vs placebo group 1.33) and an improvement of rhinitis (BHC group 0.42 versus 1.38 in the placebo group) (*p* < 0.01). The benefits of treatment were evident on the second day of treatment. Hyperemia of the nasal mucosa was significantly reduced in the BHC group (*p* < 0.01). Pain persisted in 2 patients in the treatment group versus 5 patients in the placebo group. The study also investigated days lost from school; 11 days of school were lost in the treatment group and 19 days of school were lost in the placebo group. The authors concluded that the combination of amoxicillin with BHC had a positive effect on acute sinus inflammation. Treatment was well tolerated and no side effects were attributed to the drug.

### Observational studies

Observational studies have been performed on BHC since its introduction to the market. Most of these were empiric and were based on the clinical response to drug treatment.

Molina L. carried out an observational study that examined the therapeutic effect of Na-247 (the initial preparation of BHC) in addition to the standard treatment in 48 infants (age range 1 month −4 years) affected by pharyngo-bronchitis, bronchopneumonia, bronchopneumonia with tuberculosis and asthmatic bronchitis, with primary symptoms of cough and difficult breathing [45]. Na-274 was administered (tablet or linctus) at a dose of 2–4 mg every 6 or 12 h. A control group of 20 children of similar ages with similar diagnoses received specific treatment without Na-247. The evaluation considered the following parameters: time of disappearance of symptoms upon auscultation, relief of breathing difficulties and dyspnea disappearance and time to improvement. The results were evaluated as very good (improvement after 4 days of treatment observed in 24 cases) or good (improvement from day 5–8 observed in 18 cases). The study reported an improvement in less than 4 days in 54.4% of the cases treated with Na-247 compared to 15% of the cases in the untreated group.

In another study, the efficacy of BHC administered at a dose of 0.5 mg/kg/day (in three doses) in 30 children (up to 14 years of age, of which 6 were <2 years old) with the predominant clinical symptoms of mucus retention and secretion in the respiratory tract was investigated by Fernandes [46]. The main diagnoses in the children were asthma (21 subjects), common cold (7 subjects) and bronchiolitis (2 subjects). Nineteen of the enrolled children also received antibiotics and/or antipyretics, sedatives and nasal decongestants. Treatment efficacy was evaluated by monitoring the children 4 consecutive times every 4 days. The evolution of asthmatic crises, pulmonary auscultation and cough were evaluated. The results were considered excellent (no asthmatic crisis within 48 h, pulmonary auscultation revealed absence of rhonchi/wheezing or cough disappeared within 8 days), good (asthma attacks reduced by 50%, pulmonary auscultation close to normal or coughing was no more wet). Excellent and good results were observed in 71.4% and 28.6% of the asthmatic children, respectively, and good results were observed in 42.8% of the common cold patients. No improvement was reported in children with bronchiolitis.

Brezina and Stachovà evaluated the therapeutic effects of 7–10 days of treatment with an age related dose of BHC by assessing clinical parameters in a group of 45 children (age range 1–8 years) with bronchitis [47]. Two children also received antibiotics and 2 other children received Alupent®. The authors reported that 4.4% of patients were lost during the study, 66.7% of the patients were cured, 17.7% of patients showed improvement and 11.2% of patients showed no improvement. Cured was defined as lung findings disappeared and children were problem free.

A study by Okamoto et al. evaluated the clinical effect of 1–2 weeks of treatment with BHC in 37 children of various ages with cough, stridor and expectoration due to various respiratory conditions [48], including bronchitis, the common cold, asthmatic bronchitis, asthma and bronchiectasis. BHC was administered at various doses ranging from 0.4-0.6 mg/kg. Previous drug treatments were continued. The intensity and frequency of cough, sputum quality, stridor, rhonchi, nasal discharge and sleep disturbance were evaluated before and after treatment. An overall assessment of the treatment was classified as markedly effective: symptoms disappeared or improved in 3–4 days of treatment (5.4% cases); effective: symptoms improved within 3–4 days and disappeared within 7 days (70.3%); moderately effective: symptoms showed at least one grade of improvement (16.2%); or ineffective: no improvement (8.1%). The sputum stubbornness and the frequency of expectoration effectiveness were 85%, whereas the improvement of cough was 90%. No side effects were reported in this study group.

Koga and Saito evaluated the efficacy of one week of treatment with BHC at a dose of 0.02 g/kg/day in three doses in 32 children (age range 3–9 years) with upper respiratory tract inflammation, acute bronchitis, bronchopneumonia and asthma [49]. A scoring system was used to evaluate the improvement of difficulty in expectorating sputum, the character and amount of sputum, cough, stridor, dyspnea, and rhonchus. General effectiveness results showed a cumulative improvement in symptoms of 23.3-43.8%. The study reported a high degree of improvement in the expectorability, character and amount of sputum in 70-80% of the cases.

### Comparative studies

In an open randomized comparative study, Camurri and Marenco evaluated the efficacy and tolerability of 10 days of treatment with BHC versus n-acetylcysteine in granule formulation in 32 children admitted to the hospital with diagnoses of acute bronchitis [50]. Patients were randomized to two matched treatment groups: the BHC group (age range 2.3 ± 10.8) received BHC at a dose of 24 mg/day and the N-acetylcysteine (NAC) group (age range 2.1 ± 11.11) received NAC at a dose of 300 mg/day. Any antibiotic treatment that may have been started before enrollment was continued throughout the study. The efficacy of treatment was evaluated by monitoring clinical parameters (fever, cough, breathlessness, quantity and quality of expectoration, ease of expectoration and auscultation of the chest) before and after treatment. These parameters were evaluated with a semi quantitative scale. The results demonstrated that the average scores gradually declined in both study groups by the end of treatment and non-significant differences were observed for almost all signs. However, the onset of the therapeutic action of BHC was faster, with a significant reduction apparent on day 2. Ease of expectoration was the only symptom that was significantly different between the two groups, in favor of the BHC group. Both drugs were well tolerated and no significant adverse effects were reported.

In an open randomized study, Azzollini et al. evaluated the efficacy and tolerability of two weeks of treatment with Sobrerol (50–100 mg twice daily) versus BHC (2–4 mg 3 times daily) in 40 children suffering from acute hypersecretory bronchopulmonary diseases (acute, asthmatic or recurrent bronchitis) [51]. A 3 day washout period occurred before patients were randomized to either the Sobrerol group (mean age of 43 + 18.8 months) or the BHC group (mean age of 41 + 16.3 months). Clinical symptoms were used to evaluate clinical efficacy. Body temperature, general clinical conditions, dyspnea, cough severity, sputum viscosity and volume, difficulty in expectoration and pathological sounds on thorax auscultation were scored before and after treatment. Tolerability was evaluated by reporting and describing any local or systemic effects. A significant reduction of body temperature (*p* < 0.05) was observed in both groups. In the Sobrerol group, a significant improvement of dyspnea, cough severity, sputum viscosity and difficulty of expectoration scores was observed (*p* < 0.05). In the BHC group, a statistically significant improvement of general clinical conditions, dyspnea and sputum viscosity was reported. The authors reported no significant overall differences between the two treatment groups. Both treatments demonstrated similar improvement rates. Drugs were well tolerated in both groups. The only reported slight side effects were: cutaneous rash (1 patent in the Sobrerol group), diarrhea (1 patient in the Sobrerol group and 1 patient in the bromhexine group), nausea and regurgitation (2 patients in the BHC group).

Boner et al. investigated the efficacy of combination therapy with cephalexin and BHC in 100 children (age range 2–13 years) with upper and lower tract infections [52]. These patients were affected with acute bronchitis, trachea-bronchitis, recurrent bronchitis, bronchopneumonia, purulent otitis and rhino-pharyngo-tonsillitis with sinusitis. Cephalexin (80–100 mg/kg/day) and BHC (0.6-0-8 mg/kg/day) were administered simultaneously three times a day for 5–12 days. Bacteriological evaluation was carried out on sputum samples, pharyngeal swabs, and aspirated tracheal or ear secretions depending on disease presentation. A score system was used to evaluate cough, temperature and sputum. Before treatment, bacterial strains (probable etiological agents) were isolated in 71 patients. The results demonstrated a complete recovery in 66% of cases and a marked improvement in 27% of cases. In 7% of cases, treatment failed. Clinical parameters showed a favorable response in patients suffering from acute infection compared to patients with recurrent infections. The only side effects registered were 3 cases of gastric intolerance that did not require the discontinuation of treatment. The authors concluded that treatment with BHC and cephalexin was beneficial in pediatric patients with respiratory tract infections.

## Discussion

Mucociliary clearance, a vital mechanism of pulmonary defense, requires the harmonization of many factors to be effective, including efficient ciliary beating, a steady mucus secretion rate, mucus rheology, and inherent transportability. Mucoactive agents are widely indicated in patients with acute and chronic broncho-pulmonary disorders associated with abnormal mucus secretion and impaired mucus transport [53]. The main purpose of mucoactive drugs is to increase the ability to expectorate sputum and/or decrease mucus hypersecretion. This indication is very broad and may refer to several diseases such as acute bronchitis, chronic bronchitis and chronic obstructive pulmonary disease. While the physiopathology of these diseases differs, the therapeutic effect of mucoactive drugs was initially erroneously thought to be mediated by a similar mechanism of action. Updated knowledge on the mucociliary complex system led to the further classification of these drugs on the basis of their action as expectorants, mucolytics, mucokinetic or mucoregulatory agents. Clinical studies of these drugs are still challenging, mainly due to a lack of objective parameters. The increased production and secretion of mucus or the variability of mucus contents and consequently the changes in the physico-chemical characteristic of mucus are difficult to evaluate in vivo [54, 55]. For example, one could think that expectorated sputum volume would be a good method for the assessment of the effectiveness of a therapeutic agent, but expectorated sputum volume relates poorly, at best, to improvements in pulmonary function or to the clinical status of the patient [5]. This is partly due to the limitation of measuring sputum volume because of patient reluctance to expectorate instead of swallowing of the secretions. The sputum volume also varies from day to day [5]. Furthermore, sputum contains various amounts of saliva and secretions from the pharynx, larynx, nose and nasopharynx as well as from the lungs.

BHC is a widely prescribed mucoactive over-the-counter drug used to treat a range of respiratory conditions, mainly conditions associated with mucus secretion disturbances. These conditions are predominantly associated with augmented inflammation and vulnerability to the development of infections. After the registration of BHC in Europe in 1963, numerous basic and clinical studies were focused on investigating the efficacy of BHC and understanding its mechanisms of action. In the following 15 years, interest in the drug gradually declined, although initial studies with BHC were of great interest to the medical community. These initial studies clearly suffer from a number of limitations and deficiencies. These studies were conducted in an era when methodological approaches and good clinical practices were not developed yet. In retrospect, these studies appear to lack a methodological approach and do not correspond to the current requirements of evidence-based medicine. Conflicting results were observed in the different endpoints due to a lack of details provided in the description of the patients enrolled in the studies. Study groups were heterogeneous and included patients with various diseases with different pathophysiological characteristics [54, 55]. Our understanding of respiratory diseases has changed remarkably; we now recognize different phenotypes and genotypes in a single disease entity. Although assessment of clinical data confirm the indication in all age groups, initial studies included patients with a wide range of ages and stratification by age group was not considered essential.

In this review, we analyzed the major studies concerning BHC. Several randomized controlled trials showed a significant effect of BHC in patients with respiratory conditions. Most of the patients in the studies were affected by chronic obstructive bronchitis [33–40], and mucus secretions seem to play a primary role in the pathogenesis of the disease. A selection bias may be present in studies from the pre computed tomography era, leading to an underestimation of the prevalence of specific conditions such as emphysema or bronchiectasis. The low number of patients included in the studies did not accurately reflect the number of individuals diagnosed with the condition [30, 34–36, 38, 40]. The mixed clinical endpoints were not comparable because of a lack of standardization of outcomes. Several studies used different generic nominal scales to assess symptoms and the overall clinical evaluation was biased by the physicians’ attitudes and practice. Spirometric measures were significantly different in a small number of placebo-controlled trials [36, 38, 39]. The multicenter Italian study [39], one of the largest studies evaluated, found a significant improvement in patients treated with BHC, although the patients exhibited a mean FEV_1_ of approximately 2 l.

BHC was shown to be safe in all studies. Studies in children although substantial, have endpoints that depend on the clinical evaluation of symptoms by physicians or patients’ descriptions of symptoms such as cough or expectoration. In most of the research reports, only gastrointestinal intolerance and headache were reported as side effects and were probably related to the dose. However, the dosage and duration of treatment varied. In light of the above findings, initial studies on BHC were ignored by current researchers. A recent review on clinical evidence for treatment of acute bronchitis referred to the placebo controlled study by Nesswetha going back to 1967 [32] was mentioned in the Cochrane review, showing a significant reduction of the proportion of patients with frequent cough occurrence following bromhexine (12 mg/d) [55].

Indeed, Cochrane reviews on acute cough and bronchiectasis [55, 56] considered 4 studies on BHC/ambroxol fulfilling the Cochrane RCT criteria, although the studies have been done decades ago. The trials assessed in the recent Cochrane review on OTC remedies for acute cough in children and adults [55] showed overall conflicting evidence for efficacy and thus did not lead to a clear recommendation for or against the effectiveness of OTC cough medications evaluated. Furthermore, a large placebo effect is often detected in studies that investigate respiratory conditions, particularly non-serious, self-limiting conditions.

Recently, the Pharmacovigilance Risk Assessment Committee (PRAC) of the European Medicines Agency analyzed the balance of benefits and risks for Ambroxol and BHC products and whether their approved indications are still positive [57].

The clinical studies performed during the development of bromhexine- and ambroxol-containing products between the 1950ies and 1980ies were considerably less standardised than would be necessary today, and would not completely fulfil contemporary requirements with regard to validated endpoints, statistical confirmation, or Good Clinical Practice (GCP). These constitute the majority of the available evidence, in particular in the indications that were first authorised (e.g. secretolytic indication). This is not unexpected considering the methodological challenges inherent to this therapeutic area and evidential standards and requirements at the time when these products were first developed. Often a large placebo effect is seen in studies investigating respiratory conditions, particularly in non-serious, self-limiting conditions. Further, the definition of the relevant clinical endpoints and the measurement of symptoms in these conditions are challenging [54]. However, this is common for all OTC products [55] showing conflicting evidence for efficacy. Nevertheless, modest but positive results were reported for ambroxol and bromhexine.

The Pharmacovigilance Risk Assessment Committee (PRAC) following their recent risk-benefit evaluation of all bromhexine/ambroxol products [57], as a consequence, concluded that the benefit-risk balance of ambroxol- and bromhexine-containing medicinal products remains favourable.

In studying the therapeutic efficacy of mucoactive drugs, we still lack well-defined endpoints to be used in clinical trials. The assessment of cough, expectoration and dyspnea are difficult [58]. Furthermore, the decline in lung function is associated with difficulty in expectorating secretions [5]. These parameters are difficult to measure as clinical endpoints and are prone to variability. In addition, we still lack long-term studies in this field. Improving mucociliary clearance over time reduces respiratory infections and halts the progression of anatomical and functional damage in the respiratory tract.

Clinical evidence from these studies appears weak due to a high degree of heterogeneity, few enrolled subjects, difficulty in comparing endpoints and a lack of modern scientific methodology. On the other hand, when considering data in support of BHC treatment, we found robust data from animal models that demonstrated that BHC was a mucoactive drug. BHC is able to influence mucus composition and in turn affects mucociliary activity and cough with rare and mild side effects.

## Conclusions

Although clinical evidence shows only modest but positive results, this does not mean that the drug’s efficacy is disputable. BHC treatment is associated with favorable improvements in mucus clearance and is indicated in various respiratory disorders with abnormal mucus secretion and impaired mucus transport. Furthermore, BHC is well tolerated and studies showed low incidence of mild side effects. Larger trials with adequate methodology are lacking in almost all OTC products and are in general required to identify when the treatment can improve the clinical outcome. Endpoints for future studies should include quality of life questionnaires to better assess the impact of treatment in the daily lives of patients.

## Abbreviations

BHC: Bromhexine
FEV_1_: Forced expiratory volume in one second
FRC: Functional residual capacity
FVC: Forced vital capacity
GP: Glycoprotein
LRTIs: Lower respiratory tract infections
MPS: Mucopolysaccharide
OTC: Over-the-counter
PEFR: Peak expiratory flow rate
PRAC: Pharmacovigilance Risk Assessment Committe
RCP: Raised cosine pulse
RCT: Randomized controlled trial
RV: Residual volume
TID: Three times a day
VAS: Visual analog scale
VC: Vital capacity
